# Association between plasma long-chain polyunsaturated n-3 fatty acids concentrations and cognitive function: findings from NHANES III

**DOI:** 10.3389/fpsyg.2024.1305570

**Published:** 2024-05-02

**Authors:** Xiaojing Li, Zijie Huang, Yueqin Tian, Xing Chen, Haidong Wu, Tong Wang

**Affiliations:** ^1^Department of Emergency, The Eighth Affiliated Hospital of Sun Yat-sen University, Shenzhen, China; ^2^Department of Rehabilitation Medicine, The Third Affiliated Hospital, Sun Yat-sen University, Guangzhou, Guangdong, China

**Keywords:** plasma long-chain n-3 polyunsaturated fatty acids, cognitive function, delayed recall test, docosahexaenoic acid, eicosapentaenoic acid

## Abstract

**Background:**

With increased life expectancy, cognitive decline has emerged as a prevalent neurodegenerative disorder.

**Objective:**

This study aimed to examine the correlation between concentrations of Plasma long-chain n-3 polyunsaturated fatty acids (LCPUFAs) and cognitive performance in elderly Americans.

**Methods:**

Data were analyzed from older adults enrolled in two NHANES cycles. Participants completed four cognitive assessments, including the Immediate Recall Test (IRT), Delayed Recall Test (DRT), Animal Fluency Test (AFT), and Digit Symbol Substitution Test (DSST). Linear regression and restricted cubic spline modeling examined associations between plasma LCPUFAs levels and cognitive test outcomes.

**Results:**

The cohort included 610 adults aged 69 years on average, 300 (49.2%) males and 310 (50.8%) females. The median LCPUFAs concentration was 309.4 μmol/L, with an interquartile range of 244.7–418.9 μmol/L. In unadjusted and adjusted generalized linear regression model analyses, circulating LCPUFAs exhibited significant positive correlations with DRT performance. No relationships were detected among those with chronic conditions (chronic heart failure, stroke, diabetes). A significant association between LCPUFAs levels and DRT scores was evident in males but not females.

**Conclusion:**

Plasma LCPUFAs concentrations were significantly associated with DRT performance in males free of chronic illnesses, including heart failure, stroke, and diabetes.

## 1 Introduction

Cognitive impairment is a common neurodegenerative disorder whose prevalence correlates with the aging of the population. As life expectancy increases, the proportion of individuals aged 65 years and older continues to rise globally, leading to an upward trajectory in the incidence of cognitive impairment. Cognitive impairment, such as Alzheimer’s disease (AD), currently afflicts an estimated 6.2 million Americans aged 65 and older and this number could reach 13.8 million by 2060 in the United States. Official death certificates recorded 121,499 AD fatalities in 2019, making it the sixth-leading cause of death in the United States and fifth-leading for Americans 65 and over (2021 Alzheimer’s disease facts and figures, 2021). According to the Chinese Longitudinal Healthy Longevity Survey, the age-adjusted prevalence of cognitive impairment climbed from 11.00% in 1998 to 11.84% in 2008, then dropped to 8.88% by 2014 in China (Kuang et al., 2020). Furthermore, the high morbidity diminishes patient and family quality of life and confers a tremendous socioeconomic burden. For instance, Alzheimer’s disease profoundly impacts the elderly well-being and health (Rajan et al., 2021). Expenditures on Alzheimer’s disease care in the United States swelled to $305 billion in 2020. As the American population ages, these costs are forecast to exceed $1 trillion by 2050 (Wong, 2020). In China, the total costs of dementia were $167.74 billion in 2015, projected to rise to $507.49 billion in 2030 and $1.89 trillion in 2050 (Jia et al., 2018). Thus, cognitive impairment constitutes a primary significant public health concern. Thus, cognitive impairment constitutes a primary significant public health concern. Given the increasingly severe epidemiological landscape of cognitive decline, identifying risk factors for the deterioration of cognitive function and implementing effective preventative and interventional measures against this disorder are critical.

Systemic inflammation may instigate disruptions in the immunological processes within the central nervous system, thereby potentially exacerbating the progression of diseases characterized by cognitive impairment. Conversely, the essential LCPUFAs, such as EPA and DHA, can modulate antioxidant signaling pathways and inflammatory processes, reduce cholesterol, stabilize platelets, and could serve as a mitigating factor against these deleterious effects (Djuricic and Calder, 2021). For instance, LCPUFAs can be incorporated into cell membrane phospholipids, altering membrane fluidity and cell signaling transduction, thereby altering membrane lipid kinetics to target immune cell membranes and modulating the dampening of immune cell activation and suppression of inflammation then suppressing the production of inflammatory mediators and reducing overall inflammation (Fan et al., 2018). The lipid DHA can reduce circulating triglycerides, inhibit lipopolysaccharide-induced inflammatory response and oxidative stress to decrease plaque inflammation and enhance plaque stability to prevent the progression of atherosclerosis and Cardiovascular Disease (Bae et al., 2023; Chong et al., 2023); and reduce inflammation and oxidation, as well as make the beneficial effects on gut microbiota to improve rheumatoid arthritis, characterized by immune inflammatory response (Nikiphorou and Philippou, 2023); Intravenous n-3 PUFA beneficially modulates the lipoxidation profile and reduces oxidative stress in COVID-19 (Pawelzik et al., 2023). EPA and DHA can also increase anti-inflammatory mediators and decrease pro-inflammatory mediators to balance inflammatory reactions in the body. For example, the omega-3 fatty acids EPA and DHA can help prevent inflammation in brain regions like the hippocampus by generating anti-inflammatory LOX and CYP450 lipid mediators, thus protecting neurons from inflammation-induced damage in illnesses such as depression (Borsini et al., 2021); In addition, LCPUFAs can activate transcription factors like PPARs and inhibit pathways such as NF-κB to exert anti-inflammatory effects (Calder, 2013). Besides, antioxidant signaling pathways and anti-inflammatory processes, EPA and DHA also help prevent and facilitate neural development, synaptic plasticity, and learning and memory abilities mainly by antagonizing enhanced GABA transmission (Liu et al., 2020). So, LCPUFAs regulate normal cognitive functions through anti-inflammatory effects, promoting neural development, synaptic plasticity, and learning and memory capabilities. Therefore, maintaining adequate levels of LCPUFAs is critical for cognitive processes and warrants further research.

Although previous studies have suggested that LCPUFAs may contribute to the prevention and treatment of neurodegenerative diseases with cognitive decline via anti-inflammatory effects, improved lipid metabolism, and maintenance of neuronal membrane integrity (Balakrishnan et al., 2021), the relationship between blood LCPUFAs level and cognitive function of the elderly has become the focus of research. Previous studies found a positive correlation between higher plasma levels of DHA, an important LCPUFAs, and better performance in short-term memory in mice (Xiao et al., 2022). This finding suggests that LCPUFAs, especially DHA, may play an important role in maintaining or improving cognitive function. But a common problem in these studies is that the sample size is relatively small. In addition, previous studies have focused on oral doses and in combination with other drugs, without blood concentrations. Therefore, the aim of this study was to investigate whether blood levels of LCPUFAs predict cognitive decline, thereby providing a basis for clinical detection of blood LCPUFAs and assessment of the need for exogenous LCPUFAs supplementation.

The National Health and Nutrition Examination Survey is a continuous, national-level health examination program in America that monitors and reflects the health profile of the overall population. This database contains detailed demographic information, biochemical measurements, dietary nutrition data, and cognitive function assessments, providing valuable resources to investigate diet-cognition relationships. Few studies have examined the interrelation between LCPUFAs levels and cognitive function using the NHANES dataset. Further research leveraging this dataset is warranted to elucidate the impacts of LCPUFAs on cognitive health. This study aims to utilize the information in the NHANES database and apply statistical methods to analyze the relationship between blood LCPUFAs and cognitive test performance in a representative subset of American grown-ups. The findings may provide population-level evidence for the cognitive protective effects of LCPUFAs and scientific support for preventing cognitive impairment.

## 2 Methods

### 2.1 Study population and data sources

The National Health and Nutrition Examination Survey is a substantial, elaborate survey performed across various levels by the Centers for Disease Control and Prevention (CDC). It studies non-institutionalized Americans to characterize health and nutrition across the United States. The National Health and Nutrition Examination Survey protocols received clearance from the Research Ethics Review Board of the National Center for Health Statistics, and written informed consent was secured from every volunteer before participation. Data were harvested chiefly from the 2011–2012 and 2013–2014 National Health and Nutrition Examination Survey cycles. Participants ≥60 years old undergoing cognitive testing and providing blood samples for n-3 fatty acid assay were eligible for inclusion, yielding 610 older adults in the final study population after eliminating cases with incomplete data.

### 2.2 Plasma LCPUFAs concentrations

Plasma fatty acid concentrations were quantified in a subset (n = 610) of samples obtained from adults aged ≥60 years participating in the NHANES. Plasma was collected following an 8-h overnight fast. Fatty acid content was measured using an optimized version of the methodology described by Lagerstedt et al. Briefly, 100 μL plasma aliquots were supplemented with 100 μL of a mixture containing 11 stable isotope-labeled internal fatty acid standards to enable recovery assessments. Sequential acid and base hydrolysis liberated esterified fatty acids from plasma lipids (e.g., triglycerides, phospholipids, cholesteryl esters). Re-acidification enabled the extraction of total fatty acids and internal standards from the matrix. Extracts were derivatized to pentafluorobenzyl (PFB) esters with triethylamine and reconstituted in hexane. Capillary gas chromatography and electron capture negative ion mass spectrometry were utilized to resolve the perfluoroalkyl ester-fatty acid derivatives. Analyte recovery was evaluated for each fatty acid class based on appropriate internal standards. Results were adjusted accordingly.

### 2.3 Cognitive evaluation

Trained interviewers administered a cognitive test battery encompassing the Consortium to Establish a Registry for Alzheimer’s Disease (CERAD) Word Learning test, Animal Fluency test, and the Digital Symbol Substitution Test (DSST) during private face-to-face interviews with NHANES participants aged ≥60 years. These tests evaluate various cognitive domains, including new verbal learning and memory (CERAD Word Learning), semantic fluency (Animal Fluency), speed of information processing, maintenance of attention, and working memory (DSST). The CERAD Word Learning test comprises three consecutive word registration trials, each involving immediate free recall of 10 semantically unrelated nouns (maximum score 30), followed by delayed recall after a post-learning interval (top score 10). During the 60-s Animal Fluency test, examinees name as many animal species as possible. The DSST pairs 9 abstract symbols with digits (1–9); examinees fill in the correct digits beneath each symbol according to a printed key (maximum score 133). Higher scores indicate better performance on all tests. Extensive validation exists across epidemiologic and clinical investigations.

### 2.4 Statistical analyses

Continuous indices were represented as mean ± standard error (SE), and dichotomous factors were denoted as weighted prevalence. Linear regression and Pearson’ ‘s chi-square tests compared participant characteristics across tertiles of plasma LCPUFAs concentrations. Restricted cubic spline modeling was leveraged to explore plausible non-linear correlations between continuously modeled LCPUFAs and performances on cognitive tests. Linear regression models evaluated crude and adjusted associations between LCPUFAs and cognition. Adjustments comprised: (1) demographics (age, sex) and body mass index (BMI); (2) demographics, BMI, and chronic diseases (hypertension, heart failure, coronary artery disease, stroke, cancer). Stratified analyses were performed by sex, smoking status, and presence/absence of chronic diseases. Analyses were conducted using R software (v4.1.1) with two-tailed *p* ≤ 0.05 defining statistical significance. To visualize the results, a forest plot was generated using the ‘rms’ package in R software. For each included predictor, the forest plot depicts the effect size estimate (OR) as a square and the 95% confidence interval (CI) as a horizontal line. To develop a prognostic nomogram that can be broadly utilized in primary care settings with a minimal number of key predictors, we then identified the most salient features to incorporate into the predictive model.

## 3 Results

### 3.1 Study cohort

**Figure 1:**
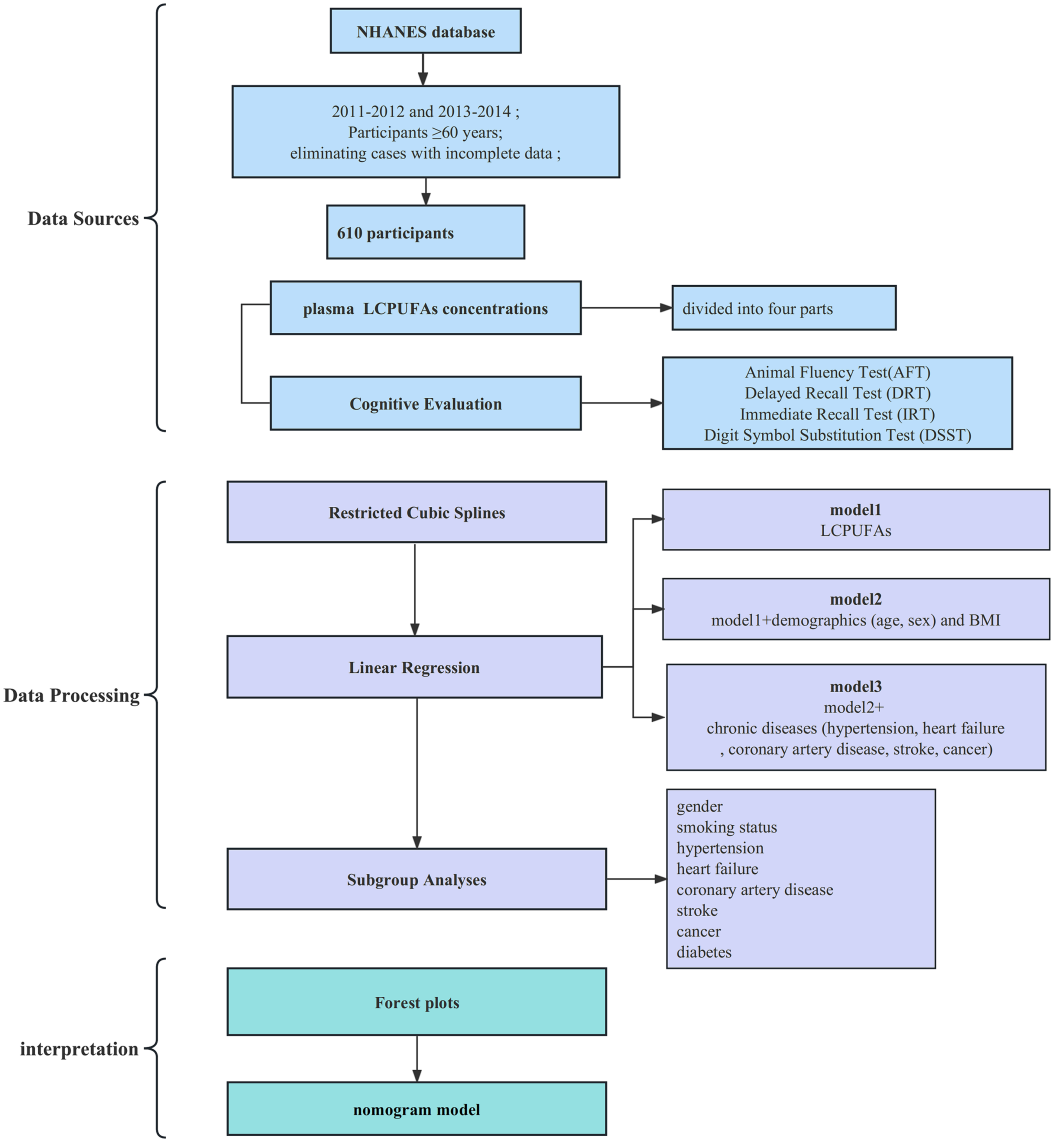
Flowchart.

The analytic sample comprised 610 adults with a mean age of 69 (49.2% male, 50.8% female). The median plasma LCPUFAs concentration was 309.4 μmol/L (interquartile range 244.7 μmol/L-418.9 μmol/L). Participants were stratified into quartiles by LCPUFAs status. The study selection process is outlined in a flow diagram (Figure 1).

### 3.2 Baseline characteristics

No significant between-quartile differences existed for age, education, marital status, family income, or income-to-poverty ratio. However, the proportion of females was higher in Q1 than in Q4 (*p* < 0.05). Additionally, Q1 had a more significant percentage of U.S born participants versus Q4 (*p* < 0.05). Regarding lifestyle factors, more non-smokers were present in Q1 than in Q4 (*p* < 0.05). Hypertension, arthritis, congestive heart failure, coronary artery disease, stroke, cancer, and diabetes prevalence did not significantly differ by quartile. Scores on AFT, DRT, IRT, and DSST showed a progressive increase across ascending quartiles, with significant between-quartile differences observed for DRT, IRT, and DSST (Table 1).

**Table 1:** Characteristics of the participants by plasma LCPUFAs level quartile.

**Variables**	**Levels**	**T1 (*N* = 153)**	**T2 (*N* = 152)**	**T3 (*N* = 152)**	**T4 (*N* = 153)**	***p***
Gender (%)	Male	99 (64.7%)	81 (53.3%)	69 (45.4%)	51 (33.3%)	<0.001
	Female	54 (35.3%)	71 (46.7%)	83 (54.6%)	102 (66.7%)	
Age(years)	Median (IQR)	69.0 [63.0,77.0]	69.0 [63.0,77.0]	68.5 [64.0,73.0]	69.0 [64.0,75.0]	0.338
Race (%)	Mexican American	22 (14.4%)	12 (7.9%)	8 (5.3%)	2 (1.3%)	<0.001
	Other Hispanic	16 (10.5%)	21 (13.8%)	26 (17.1%)	14 (9.2%)	
	Non-Hispanic White	75 (49%)	71 (46.7%)	69 (45.4%)	72 (47.1%)	
	Non-Hispanic Black	32 (20.9%)	34 (22.4%)	34 (22.4%)	34 (22.2%)	
	Other Race - Including Multi-Racial	8 (5.2%)	14 (9.2%)	15 (9.9%)	31 (20.3%)	
Country of birth (%)	Born in 50 US states or Washington, DC	129 (84.3%)	120 (78.9%)	102 (67.1%)	106 (69.3%)	0.005
	Others	24 (15.7%)	32 (21.1%)	50 (32.9%)	46 (30.1%)	
	Refused	0 (0%)	0 (0%)	0 (0%)	1 (0.7%)	
Education level (%)	Less than 9th grade	25 (16.3%)	20 (13.2%)	18 (11.8%)	16 (10.5%)	0.086
	9-11th grade (Includes 12th grade with no diploma)	24 (15.7%)	18 (11.8%)	25 (16.4%)	20 (13.1%)	
	High school graduate/GED or equivalent	39 (25.5%)	26 (17.1%)	39 (25.7%)	36 (23.5%)	
	Some college or AA degree	38 (24.8%)	54 (35.5%)	43 (28.3%)	35 (22.9%)	
	College graduate or above	27 (17.6%)	34 (22.4%)	27 (17.8%)	46 (30.1%)	
Marital status (%)	Married	99 (64.7%)	82 (53.9%)	98 (64.5%)	90 (58.8%)	0.636
	Widowed	24 (15.7%)	26 (17.1%)	26 (17.1%)	32 (20.9%)	
	Divorced	16 (10.5%)	23 (15.1%)	13 (8.6%)	15 (9.8%)	
	Separated	3 (2%)	5 (3.3%)	4 (2.6%)	2 (1.3%)	
	Never married	6 (3.9%)	10 (6.6%)	8 (5.3%)	11 (7.2%)	
	Living with partner	5 (3.3%)	6 (3.9%)	3 (2%)	2 (1.3%)	
	Refused	0 (0%)	0 (0%)	0 (0%)	1 (0.7%)	
Annual household income		7.0 [5.0,10.0]	8.0 [4.5,14.0]	7.0 [5.0,12.0]	8.0 [6.0,14.0]	0.183
Family income		7.0 [4.0,10.0]	7.0 [4.0,13.0]	7.0 [5.0,12.0]	8.0 [6.0,14.0]	0.161
Family income to poverty line ratio(%)		1.9 [1.1,3.4]	2.3 [1.2,4.7]	2.3 [1.2,4.0]	2.8 [1.2,4.5]	0.108
Body weight (kg)		80.8 [69.4,93.0]	79.3 [70.0,91.9]	79.7 [65.7,91.3]	73.1 [62.0,85.8]	<0.001
Standing height (cm)		166.7 [160.9,172.7]	166.7 [160.6,172.8]	164.4 [159.1,172.0]	160.8 [156.1,170.1]	<0.001
Arm Circumference (cm)		32.3 [30.0,35.7]	33.6 [30.2,36.1]	32.8 [30.0,35.5]	31.1 [29.0,34.6]	0.006
Waist Circumference (cm)		101.8 [94.7,110.2]	102.4 [95.8,112.0]	102.3 [93.2,111.6]	96.8 [87.2,107.2]	0.001
High blood pressure (%)	Yes	98 (64.1%)	88 (57.9%)	100 (65.8%)	92 (60.1%)	0.466
	No	54 (35.3%)	64 (42.1%)	52 (34.2%)	61 (39.9%)	
	Do not know	1 (0.7%)	0 (0%)	0 (0%)	0 (0%)	
Arthritis (%)	Yes	70 (45.8%)	79 (52%)	63 (41.4%)	67 (43.8%)	0.321
	No	83 (54.2%)	72 (47.4%)	89 (58.6%)	86 (56.2%)	
	Do not know	0 (0%)	1 (0.7%)	0 (0%)	0 (0%)	
Congestive heart failure (%)	Yes	17 (11.1%)	10 (6.6%)	10 (6.6%)	11 (7.2%)	0.547
	No	136 (88.9%)	141 (92.8%)	142 (93.4%)	141 (92.2%)	
	Do not know	0 (0%)	1 (0.7%)	0 (0%)	1 (0.7%)	
Coronary heart disease (%)	Yes	21 (13.7%)	15 (9.9%)	10 (6.6%)	11 (7.2%)	0.392
	No	131 (85.6%)	136 (89.5%)	141 (92.8%)	140 (91.5%)	
	Do not know	1 (0.7%)	1 (0.7%)	1 (0.7%)	2 (1.3%)	
Stroke (%)	Yes	19 (12.4%)	9 (5.9%)	7 (4.6%)	8 (5.2%)	0.084
	No	134 (87.6%)	142 (93.4%)	144 (94.7%)	145 (94.8%)	
	Do not know	0 (0%)	1 (0.7%)	1 (0.7%)	0 (0%)	
Cancer or malignancy (%)	Yes	27 (17.6%)	22 (14.5%)	30 (19.7%)	33 (21.6%)	0.452
	No	126 (82.4%)	129 (84.9%)	122 (80.3%)	120 (78.4%)	
	Do not know	0 (0%)	1 (0.7%)	0 (0%)	0 (0%)	
Diabetes (%)	Yes	43 (28.1%)	40 (26.3%)	37 (24.3%)	31 (20.3%)	0.695
	No	105 (68.6%)	108 (71.1%)	111 (73%)	117 (76.5%)	
	Borderline	5 (3.3%)	3 (2%)	4 (2.6%)	5 (3.3%)	
	Do not know	0 (0%)	1 (0.7%)	0 (0%)	0 (0%)	
Current smoker (%)	Every day	25 (16.3%)	16 (10.5%)	15 (9.9%)	7 (4.6%)	0.009
	Some days	4 (2.6%)	1 (0.7%)	4 (2.6%)	0 (0%)	
	Not at all	62 (40.5%)	66 (43.4%)	54 (35.5%)	59 (38.6%)	
	Missing	62 (40.5%)	69 (45.4%)	79 (52%)	87 (56.9%)	
DHA (μmol/L)		111.0 [92.0,126.0]	152.5 [137.5,168.5]	200.5 [179.0,224.0]	297.0 [257.0,357.0]	<0.001
DPA(μmol/L)		38.9 [33.6,45.6]	48.8 [42.6,56.0]	57.6 [49.0,64.6]	71.2 [58.8,85.8]	<0.001
DPA(μmol/L)		17.9 [13.6,22.1]	21.1 [17.1,25.5]	20.9 [15.8,28.5]	17.6 [11.6,24.8]	<0.001
EPA (μmol/L)		33.9 [27.0,42.3]	49.0 [39.9,62.3]	77.5 [60.1,97.8]	144.0 [109.0,203.0]	<0.001
LCPUFAs (μmol/L)		207.0 [183.5,224.5]	277.0 [260.5,290.4]	358.9 [333.9,389.4]	529.2 [449.9,656.0]	<0.001
DRT		5.0 [4.0,7.0]	6.0 [4.0,7.0]	6.0 [4.0,8.0]	6.0 [5.0,8.0]	0.003
AFT		15.0 [12.0,20.0]	16.0 [13.0,20.0]	16.0 [12.5,21.0]	16.0 [13.0,19.0]	0.469
DSST		41.0 [30.0,55.0]	47.0 [33.5,59.5]	46.5 [32.0,60.5]	46.0 [35.0,62.0]	0.028
IRT		17.0 [15.0,20.0]	18.0 [15.0,22.0]	18.5 [15.0,22.0]	20.0 [16.0,22.0]	0.005

### 3.3 Association between LCPUFAs and cognitive functions with restricted cubic splines and linear regression analysis

#### 3.3.1 IRT

**Figure 2:**
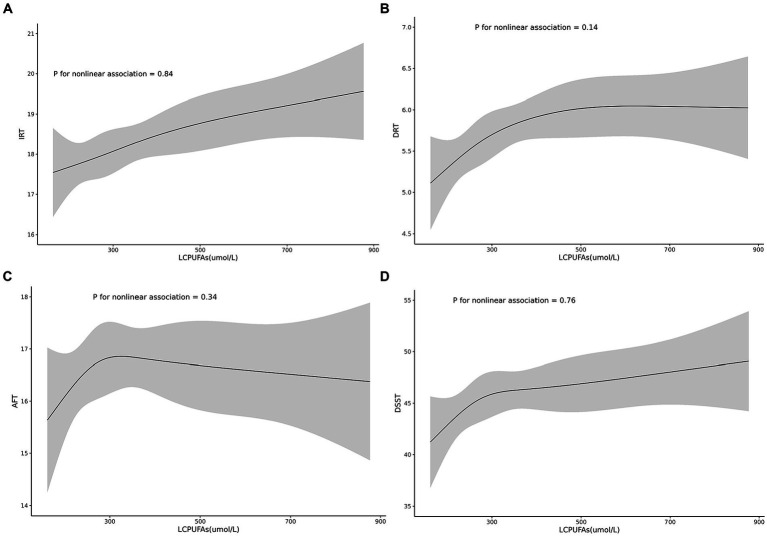
Shows the non-linear associations between the risk of cognitive function and the levels of the LCPUFAs tested for all participants. Associations of the levels of the LCPUFAs with IRT **(A)**, DRT **(B)**, AFT **(C),** and DSST **(D)**.

**Figure 3:**
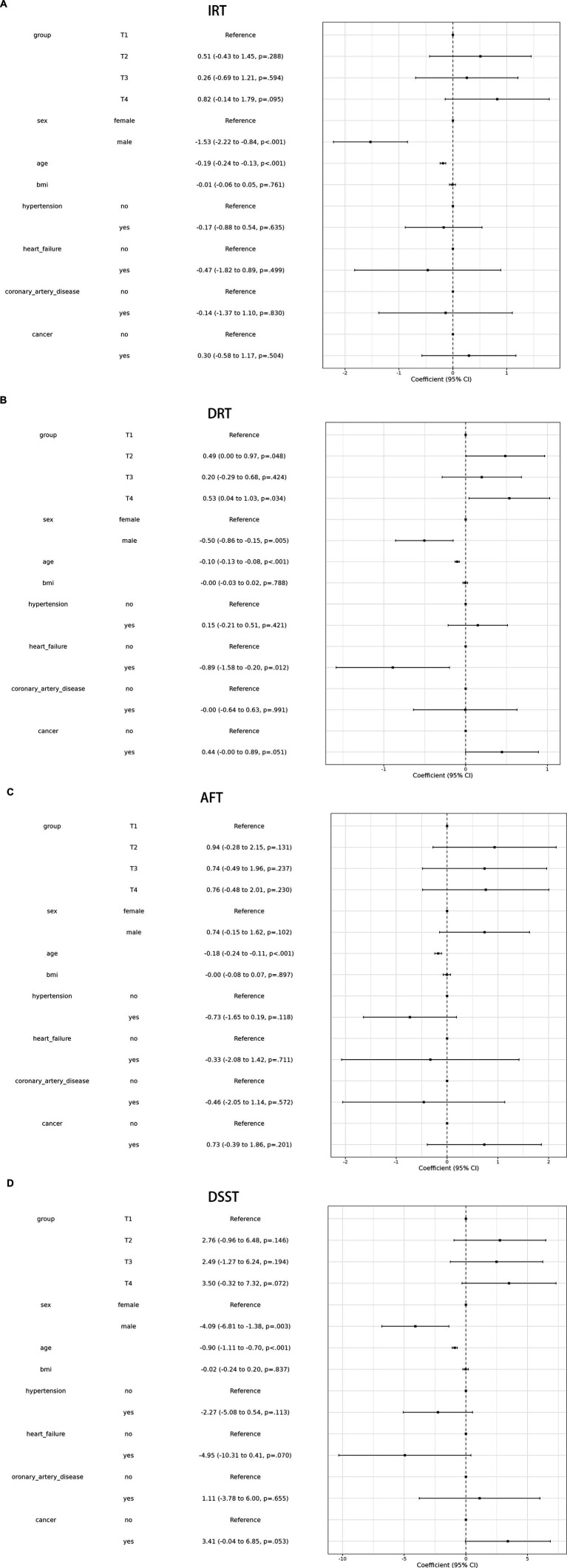
Shows the forest plot of IRT **(A)**, DRT **(B)**, AFT **(C)**, and DSST **(D)**.

A linear association between LCPUFAs and IRT was observed from Restricted cubic splines (Figure 2A, *p* = 0.84). However, in multivariable linear regression analysis, adjusted by sex, race, BMI, and chronic diseases hypertension, heart failure, coronary artery disease, stroke, cancer, the result found that the level of LCPUFAs was not associated with IRT (Table 2). As shown in the forest plot, compared with the reference group (Q1), the ORs of LCPUFAs on IRT were 0.51 (95% CI, −0.43 to 1.45, *p* = 0.288) in the Q2, 0.26 (95% CI, −0.69 to 1.21, *p* = 0.594) in the Q3, and 0.82 (95% CI, −0.14 to 1.79, *p* = 0.095) in the Q4 (Figure 3A). Among other covariates, IRT was lower in men compared with women (OR = -1.53, 95% CI, −2.22 to −0.84, *p* < 0.001); and with increasing age, IRT was lower (OR = -0.19, 95%CI, −2.24 to −0.13, *p* < 0.001). In contrast, BMI, hypertension, heart failure, coronary artery disease, and tumor were not significantly associated with IRT.

**Table 2:** The association between LCPUFAs and cognitive function.

	**Model1**		**Model2**		**Model3**	
	**OR(95%CI)**	***p***	**OR(95%CI)**	***p***	**OR(95%CI)**	***p***
AFT						
T1 (*N* = 153)	Reference		Reference		Reference	
T2 (*N* = 152)	1.07 [−0.15, 2.30]	*p* = 0.086	1.10 [−0.10, 2.31]	*p* = 0.073	0.96 [−0.25, 2.17]	*p* = 0.119
T3 (*N* = 152)	0.94 [−0.29, 2.17]	*p* = 0.132	0.84 [−0.38, 2.06]	*p* = 0.178	0.79 [−0.42, 2.01]	*p* = 0.201
T4 (*N* = 153)	0.75 [−0.47, 1.98]	*p* = 0.228	0.84 [−0.40, 2.08]	*p* = 0.182	0.80 [−0.44, 2.04]	*p* = 0.204
IRT						
T1 (*N* = 153)	Reference		Reference		Reference	
T2 (*N* = 152)	0.81 [−0.17, 1.78]	*p* = 0.106	0.60 [−0.33, 1.53]	*p* = 0.206	0.52 [−0.42, 1.46]	*p* = 0.278
T3 (*N* = 152)	1.02 [0.04, 1.99]	*p* = 0.042	0.38 [−0.56, 1.32]	*p* = 0.431	0.30 [−0.65, 1.24]	*p* = 0.540
T4 (*N* = 153)	1.52 [0.55, 2.50]	*p* = 0.002	0.93 [−0.02, 1.89]	*p* = 0.055	0.87 [−0.09, 1.83]	*p* = 0.077
DRT						
T1 (*N* = 153)	Reference		Reference		Reference	
T2 (*N* = 152)	0.59 [0.09, 1.09]	*p* = 0.022	0.51 [0.03, 0.99]	*p* = 0.038	0.48 [−0.00, 0.96]	*p* = 0.052
T3 (*N* = 152)	0.53 [0.03, 1.03]	*p* = 0.039	0.28 [−0.21, 0.76]	*p* = 0.262	0.21 [−0.28, 0.69]	*p* = 0.404
T4 (*N* = 153)	0.83 [0.33, 1.33]	*p* = 0.001	0.61 [0.12, 1.10]	*p* = 0.016	0.54 [0.05, 1.03]	*p* = 0.032
DSST						
T1 (*N* = 153)	Reference		Reference		Reference	
T2 (*N* = 152)	4.07 [0.15, 8.00]	*p* = 0.042	24.14 [0.60–970.50]	*p* = 0.092	2.67 [−1.04, 6.38]	*p* = 0.157
T3 (*N* = 152)	4.92 [1.00, 8.84]	*p* = 0.014	13.87 [0.33–580.77]	*p* = 0.168	2.35 [−1.39, 6.08]	*p* = 0.218
T4 (*N* = 153)	5.61 [1.70, 9.53]	*p* = 0.005	43.26 [0.98–1915.09]	*p* = 0.052	3.46 [−0.34, 7.26]	*p* = 0.074

Model 1 was unadjusted; model 2 was adjusted for sex, race, and BMI; model 3 was adjusted for sex, race, BMI, and chronic diseases (hypertension, heart failure, coronary artery disease, stroke, cancer).

#### 3.3.2 DRT

It was observed by Restricted cubic splines that there was a linear association between LCPUFAs and DRT (*p* = 0.14) (Figure 2B). In multivariable linear regression analysis, after adjusting for sex, race, BMI, and chronic diseases (hypertension, heart failure, coronary artery disease, stroke, cancer), the result found significant correlation between the level of LCPUFAs and DRT (Table 2). In the stratified analyses, DRT was lower in men compared with women (OR = −0.50, 95% CI, −0.86 to −0.15, *p* = 0.005); and DRT was lower in aged participants (OR = −0.10, 95% CI, −0.13 to −0.08, *p* < 0.001) and patients with heart failure (OR = −0.89, 95% CI, −1.58 to −0.20, *p* = 0.012). However, BMI, hypertension, coronary artery disease, and tumor were not significantly correlated to DRT (Figure 3B).

#### 3.3.3 AFT

Restricted cubic splines revealed a linear association between LCPUFAs and AFT (*p* = 0.34) (Figure 2C). However, in multivariable linear regression analysis, adjusted for sex, race, BMI, and chronic diseases (hypertension, heart failure, coronary artery disease, stroke, cancer), the result showed that the level of LCPUFAs had no significant influence on AFT (Table 2). In comparison with the reference group (Q1), the ORs of LCPUFAs on AFT were 0.94 (95% CI, −0.28–2.15, *p* = 0.131) in the Q2, 0.74 (95% CI, −0.49–1.96, *p* = 0.237) in the Q3, and 0.76 in the Q4 (95% CI, −0.48–2.01, *p* = 0.230). Among other covariates, as age increased AFT went lower (OR = –0.18, 95%CI, −0.24 to −0.11, *p* < 0.001). However, neither age, BMI, heart failure, hypertension, coronary artery disease, nor tumor displayed a significant correlation with AFT (Figure 3C).

#### 3.3.4 DDST

Restricted cubic splines suggested a linear association between LCPUFAs and DDST (*p* = 0.76) (Figure 2D). But in multivariable linear regression analysis, after adjustment for sex, race, BMI, and chronic diseases (hypertension, heart failure, coronary artery disease, stroke, cancer), the result found no significant correlation between the level of LCPUFAs and DDST (Table 2). When compared to the reference group (Q1), the ORs of LCPUFAs on DDST were 2.76 (95% CI, −0.96 to 6.48, *p* = 0.146) in Q2, 2.49 (95% CI, −1.27–6.24, *p* = 0.194) in Q3, and 3.50 in Q4 (95% CI, −0.32 to 7.32, *p* = 0.072). Among other covariates, DDST was lower in men compared with women (OR = –4.09, 95% CI, −6.81 to −1.38, *p* = 0.003); and older individuals showed lower DDST (OR = -0.90, 95% CI, −1.11 to 0.70, *p* < 0.001). In contrast, there was no significant association between BMI, hypertension, heart failure, coronary artery disease, and tumor with DDST (Figure 3D).

### 3.4 Subgroup analyses

Stratified regression models were performed by gender, smoking status, and presence/absence of hypertension, heart failure, coronary artery disease, stroke, cancer, and diabetes (Table 3). No modification by smoking status, hypertension, heart failure, or cancer was observed for the LCPUFAs-DRT association. The results showed that while there are no statistical differences found between males and females in terms of race, BMI, education, and employment growth, significant variances were observed in plasma levels of LCPUFAs and DRT performances. A more detailed analysis revealed that a significant positive relationship between LCPUFAs and DRT was evident in males (OR = 0.78, 95% CI, 0.05 to 1.50, *p* = 0.035) but not females (OR = 0.50, 95% CI, −0.22 to 1.22, *p* = 0.172). Besides, women consume less energy, carbohydrates, proteins and fats daily than men. In particular, men’s daily intake of saturated fatty acids and cholesterol, both of which have been recognized as harmful to human health, is significantly higher. As for working situation, our analysis showed that, on the one hand, 23.23% of men were self-employed in their own business, professional practice or farm, while only 11.76% of women were engaged in these. On the other hand, 16.16% of men were government employees, while 26.47% of women worked in the government department (Table 4). Moreover, the significant LCPUFAs-DRT association remained only among those without chronic conditions, including heart failure, stroke, and diabetes. No significant association was detected in those with these comorbidities (Table 3).

**Table 3:** Subgroup analysis for the association between LCPUFAs and cognitive function.

		**T1** **183.5–224.5 μmol/L**	**T2** **260.5, 290.4 μmol/L**	**T3** **333.9–389.4 μmol/L**	**T4** **449.9–656.0 μmol/L**
Sex					
Male		Reference	0.27 (−0.35, 0.90), *p* = 0.391	−0.15 (−0.80, 0.51), *p* = 0.661	0.78 (0.05, 1.50), *p* = 0.035
Female		Reference	0.66 (−0.11, 1.42), *p* = 0.091	0.47 (−0.28, 1.21), *p* = 0.217	0.50 (−0.22, 1.22), *p* = 0.172
Smoking					
	Yes	Reference	0.22 (−1.16, 1.60), *p* = 0.750	−0.10 (−1.47, 1.28), *p* = 0.886	0.77 (−1.15, 2.69), *p* = 0.424
	No	Reference	0.09 (−0.62, 0.81), *p* = 0.797	−0.01 (−0.77, 0.76), *p* = 0.987	0.38 (−0.37, 1.13), *p* = 0.324
High blood pressure					
	Yes	Reference	0.66 (0.07, 1.26), *p* = 0.028	0.43 (−0.15, 1.00), *p* = 0.147	0.47 (−0.13, 1.06), *p* = 0.125
	No	Reference	0.19 (−0.65, 1.03), *p* = 0.660	−0.27 (−1.16, 0.61), *p* = 0.545	0.47 (−0.41, 1.36), *p* = 0.293
Congestive heart failure					
	Yes	Reference	0.93 (−0.96, 2.82), *p* = 0.326	0.70 (−1.17, 2.57), *p* = 0.452	1.35 (−0.56, 3.25), *p* = 0.160
	No	Reference	0.42 (−0.08, 0.92), *p* = 0.103	0.13 (−0.38, 0.63), *p* = 0.626	0.48 (−0.03, 1.00), *p* = 0.066
Coronary heart disease					
	Yes	Reference	0.83 (−0.57, 2.24), *p* = 0.237	0.20 (−1.39, 1.79), *p* = 0.799	−0.10 (−1.74, 1.53), *p* = 0.899
	No	Reference	0.45 (−0.07, 0.96), *p* = 0.088	0.20 (−0.32, 0.71), *p* = 0.450	0.60 (0.08, 1.13), *p* = 0.023
Stroke					
	Yes	Reference	0.11 (−1.72, 1.93), *p* = 0.905	−0.02 (−2.04, 2.01), *p* = 0.988	−0.32 (−2.25, 1.60), *p* = 0.734
	No	Reference	0.52 (0.01, 1.02), *p* = 0.045	0.20 (−0.31, 0.70), *p* = 0.448	0.58 (0.06, 1.10), *p* = 0.030
Cancer					
	Yes	Reference	0.59 (−0.71, 1.88), *p* = 0.371	0.67 (−0.55, 1.89), *p* = 0.278	0.54 (−0.71, 1.79), *p* = 0.391
	No	Reference	0.42 (−0.11, 0.94), *p* = 0.118	0.07 (−0.46, 0.61), *p* = 0.791	0.50 (−0.04, 1.05), *p* = 0.070
Diabetes					
	Yes	Reference	1.08 (0.10, 2.05), *p* = 0.031	0.29 (−0.71, 1.28), *p* = 0.571	−0.08 (−1.16, 1.00), *p* = 0.886
	No	Reference	0.27 (−0.29, 0.83), *p* = 0.349	0.12 (−0.45, 0.68), *p* = 0.686	0.60 (0.04, 1.17), *p* = 0.037

**Table 4:** Characteristics of the participants by sex.

Variables	Total (*n* = 610)	Male (*n* = 300)	Female (*n* = 310)	Statistic	*p*
BMI, Mean ± SD	29.28 ± 6.35	29.23 ± 5.61	29.32 ± 6.99	*t* = −0.17	0.863
Race, *n* (%)				χ^2^ = 1.37	0.849
Mexican American	44 (7.21)	23 (7.67)	21 (6.77)		
Other Hispanic	77 (12.62)	36 (12.00)	41 (13.23)		
Non-Hispanic White	287 (47.05)	137 (45.67)	150 (48.39)		
Non-Hispanic Black	134 (21.97)	67 (22.33)	67 (21.61)		
Other Race – Including Multi-Racial	68 (11.15)	37 (12.33)	31 (10.00)		
Country of birth, *n* (%)				–	0.779
Born in 50 US states or Washington, DC	457 (74.92)	223 (74.33)	234 (75.48)		
Others	152 (24.92)	76 (25.33)	76 (24.52)		
Refused	1 (0.16)	1 (0.33)	0 (0.00)		
Education, n(%)				χ^2^ = 0.98	0.912
Less than 9th grade	79 (12.95)	37 (12.33)	42 (13.55)		
9-11th grade (Includes 12th grade with no diploma)	87 (14.26)	44 (14.67)	43 (13.87)		
High school graduate/GED or equivalent	140 (22.95)	66 (22.00)	74 (23.87)		
Some college or AA degree	170 (27.87)	83 (27.67)	87 (28.06)		
College graduate or above	134 (21.97)	70 (23.33)	64 (20.65)		
Marital Status, *n* (%)				χ^2^ = 72.36	**<0.001**
Married	369 (60.49)	221 (73.67)	148 (47.74)		
Widowed	108 (17.70)	17 (5.67)	91 (29.35)		
Divorced	67 (10.98)	32 (10.67)	35 (11.29)		
Separated	14 (2.30)	4 (1.33)	10 (3.23)		
Never married	35 (5.74)	14 (4.67)	21 (6.77)		
Living with partner	16 (2.62)	11 (3.67)	5 (1.61)		
Refused	1 (0.16)	1 (0.33)	0 (0.00)		
DHA, Mean ± SD	195.01 ± 94.21	173.81 ± 77.69	215.52 ± 103.89	*t* = −5.63	**<0.001**
DP3, Mean ± SD	56.06 ± 20.26	52.75 ± 18.26	59.26 ± 21.58	*t* = −4.02	**<0.001**
DP6, Mean ± SD	20.31 ± 8.55	19.36 ± 8.07	21.24 ± 8.92	*t* = −2.73	**0.006**
EPA, Mean ± SD	86.15 ± 86.73	72.91 ± 59.65	98.95 ± 105.10	*t* = −3.78	**<0.001**
DRT, Mean ± SD	5.70 ± 2.24	5.40 ± 2.15	5.98 ± 2.30	*t* = −3.26	**0.001**
AFT, Mean ± SD	16.57 ± 5.45	16.91 ± 5.71	16.25 ± 5.18	*t* = 1.51	0.132
DSST, Mean ± SD	45.53 ± 17.54	43.49 ± 15.96	47.51 ± 18.76	*t* = −2.85	**0.005**
IRT, Mean ± SD	18.23 ± 4.37	17.38 ± 4.24	19.06 ± 4.34	*t* = −4.84	**<0.001**
Dietary habits (per day)					
Energy (kcal), Mean ± SD	1834.66 ± 751.09	2088.97 ± 832.96	1581.24 ± 554.78	*t* = 8.54	**<0.001**
Protein (gm), Mean ± SD	72.30 ± 33.17	82.57 ± 36.10	62.06 ± 26.28	*t* = 7.73	**<0.001**
Carbohydrate (gm), Mean ± SD	227.08 ± 99.86	255.30 ± 111.72	198.97 ± 76.93	*t* = 6.99	**<0.001**
Total sugars (gm), Mean ± SD	97.62 ± 61.17	107.78 ± 69.49	87.50 ± 49.64	*t* = 4.00	**<0.001**
Dietary fiber (gm), Mean ± SD	17.39 ± 11.18	19.03 ± 13.34	15.76 ± 8.20	*t* = 3.51	**<0.001**
Total fat (gm), Mean ± SD	69.55 ± 37.49	79.59 ± 41.52	59.54 ± 29.86	*t* = 6.60	**<0.001**
Total saturated fatty acids (gm), Mean ± SD	21.99 ± 12.90	24.78 ± 13.86	19.21 ± 11.22	*t* = 5.26	**<0.001**
Total monounsaturated fatty acids (gm), Mean ± SD	25.27 ± 15.11	29.58 ± 17.12	20.97 ± 11.29	*t* = 7.06	**<0.001**
Total polyunsaturated fatty acids (gm), Mean ± SD	16.76 ± 10.86	18.90 ± 11.86	14.62 ± 9.31	*t* = 4.78	**<0.001**
Cholesterol (mg), Mean ± SD	256.78 ± 195.68	299.75 ± 219.90	213.96 ± 157.19	*t* = 5.34	**<0.001**
Job/work situation, *n* (%)				–	**0.022**
An employee of a private company, business, or individual for wages, salary, or commission.	99 (59.28)	60 (60.61)	39 (57.35)		
Self-employed in own business, professional practice or farm.	31 (18.56)	23 (23.23)	8 (11.76)		
Government employee	34 (20.36)	16 (16.16)	18 (26.47)		
Miss	3 (1.80)	0 (0.00)	3 (4.41)		

*t, t*-test; −, Fisher exact; SD, standard deviation. DHA, docosahexaenoic acid (22,6n-3); DP3, docosapentaenoic acid (22,5n-3); DP6, docosapentaenoic acid (22,5n-6); EPA, eicosapentaenoic acid; IRT, the immediate recall test; DRT, delayed recall test; AFT, animal fluency test; DSST, digit symbol substitution test.

### 3.5 Forest plot and nomogram

**Figure 4:**
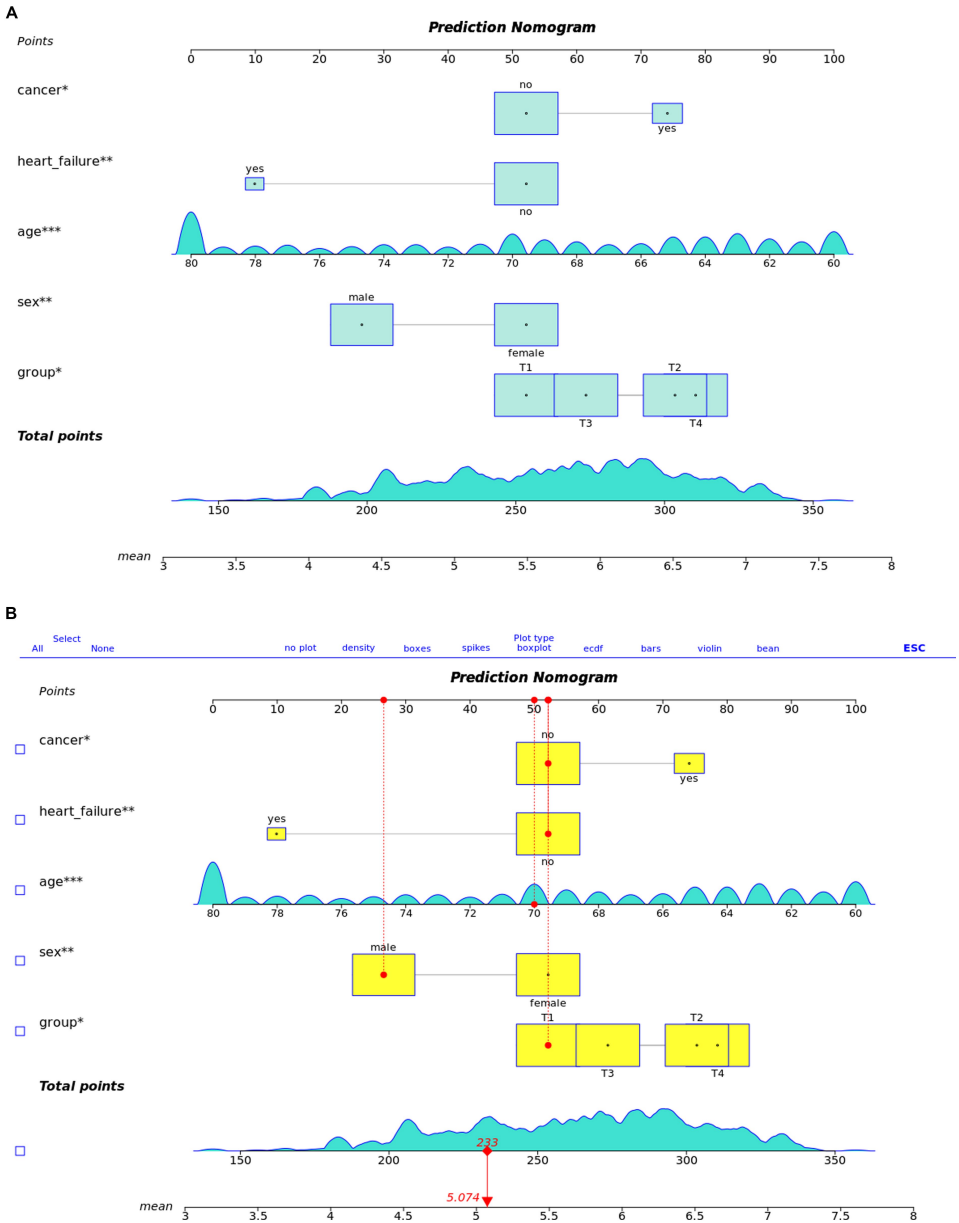
Shows the nomogram.

We utilized these key predictors to develop a prognostic nomogram for estimating the risk of cognitive impairment in older adults (Figure 4). This nomogram enables the manual calculation of a risk score for an individual.

In summary, the LCPUFAs-cognitive association differed by gender and absence of certain chronic diseases but was not modified by lifestyle factors like smoking. These findings reveal potential demographic and health-related factors influencing relationships between fatty acid status and cognitive performance.

## 4 Discussion

As life expectancy rises, cognitive decline poses an intensifying public health challenge. This large cross-sectional analysis explored associations between plasma LCPUFAs concentrations and cognitive test performance among older adults, including the AFT, DRT, IRT, and DSST. The DRT evaluates delayed verbal recall, representing the memory domain. Significant positive relationships were evident between LCPUFAs quartiles and DRT scores in unadjusted and adjusted models. Stratified analyses were undertaken by gender, smoking status, hypertension, heart failure, coronary disease, stroke, cancer, and diabetes. A significant association between higher LCPUFAs and superior DRT performance was restricted to males and those without chronic conditions, including heart failure, stroke, and diabetes; no significant relationships were observed in those with these comorbidities. In summary, higher circulating LCPUFAs related to better memory test performance in older males and chronically healthy adults in this nationally representative sample. These findings reveal demographic and health factors influencing n-3 fatty acid-cognition linkages later in life that warrant further investigation. Elucidating relationships between nutritional status and cognitive aging will contribute to developing interventions to preserve cognitive health with age.

This study identified robust positive correlations between plasma LCPUFAs levels and DRT performance, indicating a significant relationship. Echoing this finding, a prior meta-analysis highlighted that sustained high dosages (1500–2000 mg/d) of omega-3 polyunsaturated fatty acids, prominently featuring eicosapentaenoic acid, could notably alleviate cognitive decline in patients with Alzheimer’s dementia (Tseng et al., 2023). Building on this premise, extensive research has been conducted to elucidate the mechanisms through which LCPUFAs ameliorate cognitive deficits. Notably, studies have underscored that reductions in brain polyunsaturated fatty acids correlate with impaired memory functions, a phenomenon linked with diminished hippocampal and parahippocampal volumes, regions crucial for memory processing (Macaron et al., 2021). Expanding on this line of inquiry, experiments involving mice fed with DHA revealed enhanced cognitive capabilities. This improvement is attributed to DHA’s potential in mitigating amyloid deposition and promoting nerve fiber production in Alzheimer’s disease, thereby offering a plausible mechanism for its cognitive benefits (Xiao et al., 2022). In concert with DHA’s effects, EPA has likewise been shown to exert anti-inflammatory actions within the brain, influencing neurotrophic factors like BDNF, and fostering neurogenesis (Dong et al., 2018). These actions collectively illustrate how EPA and DHA contribute to neurotransmission and bolster learning and memory capabilities, thereby enhancing cognitive functions (Dighriri et al., 2022). By connecting these findings, it becomes evident that elevated levels of LCPUFAs might safeguard cognitive health via multiple neural pathways, suggesting a multifaceted approach to enhancing brain function. This evidence compellingly underscores the status of LCPUFAs as a promising modifiable factor that could significantly impact the trajectory of cognitive aging, providing a solid foundation for further research and potential therapeutic interventions.

Our analysis revealed that plasma LCPUFAs were significantly higher in females. Previous studies corroborate these findings, showing that concentrations of LCPUFAs, specifically eicosapentaenoic acid and docosahexaenoic acid, are significantly higher in females by 13 and 46%, respectively (Kitson et al., 2012). This might be rooted in the biological differences between genders, notably in the conversion rates from precursor fatty acids to LCPUFAs. Recent studies have shown that, compared to men, women have 18 and 3% higher conversion efficiencies of precursor substances to DHA and DPA, respectively, resulting in higher levels of LCPUFAs (Zhao et al., 2020). The high conversion rate may be due to the up-regulation of the expression of fatty acid desaturase 2, a Δ6-desaturase-encoding gene in human primary hepatocytes. Previous studies have shown that the activity of fatty acid desaturase 2 in female rats was higher, and the levels of newly synthesized DHA and DPA in erythrocytes of female rats increased (Kitson et al., 2012). Further, a growing body of animal model research identifies mechanisms by which sex hormones such as estrogen and progesterone interact with the synthesis of EPA and DHA. This might serve to mitigate the adverse effects exerted by low levels of LCPUFAs on cognitive function (Childs, 2020). In respect of gender difference in cognition, women exhibit verbal and semantic memory strengths that could mask the effect of low plasma concentration of LCPUFAs effects on the DRT. Episodic memory tasks entail learning target word lists across repeated trials, recalling interference lists, and short and long-term delayed recalls and recognition. Women demonstrate superior episodic memory because research indicates the right temporal lobeis larger in women than men, playing integral roles in speech processing and auditory attention, attenuating associations between DHA and delayed recall (Kljajevic, 2021). With regard to dietary habits, data revealed that women consume a low-sugar, low-fat diet daily, especially lower in harmful saturated fatty acids and cholesterol than men. This healthier diet may enable women to sustain higher plasma LCPUFAs status over time, thus potentially attenuating DRT relationships (Alkazemi, 2019). Finally, our analysis also observed that relatively more men were self-employed in their own businesses, professional practices, or farms, whereas more women were government employees. Due to the stability of work income and social status, people with higher social status and higher income tend to choose healthier diets, while low-income people tend to eat unhealthily in stores, restaurants and fast food sources (Patetta et al., 2019). This difference in eating habits may be the reason for the difference in LCPUA3 in the blood of men and women. In summary, differences in fatty acid desaturase 2 *in vivo*, cognitive biases and lifestyle factors may be intertwined, resulting in the detection of selective LCPUFAs-DRT associations in men.

Moreover, significant positive associations between circulating LCPUFAs and DRT performance were evident only among adults free of select chronic conditions, including heart failure, stroke, and diabetes. The absence of significant relationships in those with these comorbidities may be attributable to several interconnected factors. First, chronic diseases and pharmacological therapies frequently disrupt fatty acid absorption, transport, and metabolism, attenuating cognition linkages (Rink and Khanna, 2011; Stanley et al., 2012; Gaudet et al., 2015). Second, chronic inflammatory states accompanying these conditions can compromise blood–brain barrier patency and impede the delivery of fatty acids required for neuronal integrity (Pan et al., 2015). Third, the stress of living with chronic illness itself may independently worsen aspects of cognition like anxiety and depression in ways that could mask beneficial fatty acid effects (Marin et al., 2011). The complex interplay between fatty acid biology, chronic disease pathophysiology, and neurocognitive health likely alters relationships between circulating LCPUFAs and cognition in chronic comorbidities. Elucidating the biological underpinnings of this effect modification may ultimately inform strategies to extend cognitive benefits to broader populations.

Modulating LCPUFAs may represent an effective cognitive health promotion strategy based on the associations observed herein. However, certain limitations should be considered when interpreting these cross-sectional results. First, the cross-sectional design precludes causal determinations regarding relationships between circulating LCPUFAs and cognitive performance. Second, the assessment of a limited cognitive battery provides only a fractional view of overall cognition. Finally, while extensive covariate adjustment was implemented, residual confounding cannot be excluded. Large prospective studies are required to validate these findings and investigate whether LCPUFAs supplementation can improve or stabilize cognitive function over time. Notwithstanding these limitations, the results confer essential implications regarding the potential impact of optimizing LCPUFAs status on preventing and treating age-related cognitive decline. In summary, this study provides novel population-level evidence supporting the optimization of circulating LCPUFAs for maintaining cognitive health into older age. Continued efforts to obtain robust evidence in this emerging area are warranted to improve public health related to cognitive aging.

## 5 Conclusion

This cross-sectional analysis observed a significant association between plasma LCPUFAs concentrations and DRT performance among cognitively impaired individuals. Furthermore, plasma LCPUFAs levels exhibited a significant positive correlation with DRT performance in males but not females and also exhibited a significant positive correlation among adults free of select chronic conditions, including heart failure, stroke, and diabetes. Additional longitudinal analyses are imperative to garner more robust evidence. Nevertheless, these preliminary data propose that elevated circulating LCPUFAs levels may confer protection against age-associated cognitive decline.

## Data availability statement

The original contributions presented in the study are included in the article/supplementary material, further inquiries can be directed to the corresponding authors.

## Ethics statement

The studies involving humans were approved by Research Ethics Review Board of the National Center for Health Statistics. The studies were conducted in accordance with the local legislation and institutional requirements. Written informed consent for participation was not required from the participants or the participants’ legal guardians/next of kin in accordance with the national legislation and institutional requirements.

## Author contributions

XL: Conceptualization, Formal analysis, Project administration, Writing – original draft. YT: Conceptualization, Formal analysis, Investigation, Project administration, Writing – original draft. XC: Conceptualization, Formal analysis, Investigation, Project administration, Writing – original draft. HW: Conceptualization, Supervision, Writing – review & editing. TW: Conceptualization, Funding acquisition, Supervision, Writing – review & editing. ZH: Conceptualization, Formal analysis, Writing – review & editing.
